# CRP Is Transported by Monocytes and Monocyte-Derived Exosomes in the Blood of Patients with Coronary Artery Disease

**DOI:** 10.3390/biomedicines8100435

**Published:** 2020-10-19

**Authors:** Ivan Melnikov, Sergey Kozlov, Olga Saburova, Ekaterina Zubkova, Olga Guseva, Sergey Domogatsky, Tatiana Arefieva, Natalia Radyukhina, Maria Zvereva, Yuliya Avtaeva, Lyudmila Buryachkovskaya, Zufar Gabbasov

**Affiliations:** 1 National Medical Research Centre of Cardiology of the Ministry of Health of the Russian Federation, 3-rd Cherepkovskaya Street, 121552 15A Moscow, Russia; ivsgml@gmail.com (I.M.); bestofall@inbox.ru (S.K.); saburovaos@mail.ru (O.S.); ver-mishel@mail.ru (E.Z.); gusoa@mail.ru (O.G.); spdomo@yandex.ru (S.D.); areftan2@gmail.com (T.A.); info@cardioweb.ru (N.R.); zverevamd23@gmail.com (M.Z.); julia_94fs@mail.ru (Y.A.); livbur@mail.ru (L.B.); 2 State Research Center of the Russian Federation—Institute of Biomedical Problems of Russian Academy of Sciences, Khoroshevskoye Shosse, 123007 76A Moscow, Russia

**Keywords:** coronary artery disease, chronic inflammation, pentameric form of C-reactive protein, monomeric form of C-reactive protein, monocytes, microparticles, exosomes

## Abstract

The objective of this work was to study the ability of blood cells and their microparticles to transport monomeric and pentameric forms of C-reactive protein (mCRP and pCRP) in the blood of patients with coronary artery disease (CAD). Blood was obtained from 14 patients with CAD 46 ± 13 years old and 8 healthy volunteers 49 ± 13.6 years old. Blood cells and microparticles with mCRP and pCRP on their surface were detected by flow cytometry. Messenger RNA (mRNA) of CRP was extracted from peripheral blood monocytes stimulated with lipopolysaccharide (LPS) and granulocyte-macrophage colony-stimulating factor (GM-CSF). mRNA of CRP in monocytes was detected with PCR. Monocytes were predominantly pCRP-positive (92.9 ± 6.8%). mCRP was present on 22.0 ± 9.6% of monocyte-derived exosomes. mCRP-positive leukocyte-derived microparticle counts were significantly higher (8764 ± 2876/µL) in the blood of patients with CAD than in healthy volunteers (1472 ± 307/µL). LPS and GM-CSF stimulated monocytes expressed CRP mRNA transcripts levels (0.79 ± 0.73-fold), slightly lower relative to unstimulated hepatocytes of the HepG2 cell line (1.0 ± 0.6-fold), but still detectable. The ability of monocytes to transport pCRP in blood flow, and monocyte-derived exosomes to transmit mCRP, may contribute to the maintenance of chronic inflammation in CAD.

## 1. Introduction

Monomeric C-reactive protein (mCRP) is a form of C-reactive protein (CRP), which may possess pro-inflammatory properties [1]. It is formed through nonproteolytic dissociation of pCRP on membranes of activated cells and their microparticles [2,3]. The process of dissociation is described in detail elsewhere [4]. After dissociation, mCRP activates in vitro endotheliocytes, platelets, and leukocytes, and stimulates the generation of reactive oxygen species, expression of adhesion molecules, and cytokine and chemokine release [5,6,7]. mCRP is also considered a pro-angiogenic molecule. It may have a stimulatory role in neovascular formation in regions of the brain after strokes, atherosclerotic lesions, and β-amyloid plaques in Alzheimer’s disease [8,9,10]. Also, mCRP was shown to enhance thrombus formation [8,11]. It is most likely that mCRP represents a tissue-bound form of CRP, because its deposits were described in human atherosclerotic plaques, β-amyloid plaques, necrotic regions of the brain after strokes, as well as in choroid of patients with age-related macular degeneration [3,12,13].

Whether mCRP is synthesized locally in areas of inflammation or transported therein from blood flow, remains a subject of discussion. Several papers argue in favor of local CRP synthesis by macrophages. Kaplan et al. showed that monocyte-derived macrophages express CRP mRNA [13]. Dong et al. provided evidence of CRP mRNA expression by alveolar macrophages [14]. Kolb-Bachofen et al. reported mCRP expression by human peripheral blood monocytes [15]. Ciubotaru et al. described mCRP production by U937 line of macrophages [16]. 

The evidence that mCRP may be present in circulating blood is sparse. To date, it is known that mCRP can be transported in blood flow on microparticles [17,18]. Otherwise, potential pathways of mCRP transportation in circulating blood and possible delivery to areas of local inflammation are poorly studied. In this work, we studied blood cells and their microparticles to search for the possible ways of transportation of mCRP in blood flow. We also studied mCRP expression by peripheral blood monocytes, addressing the problem of local mCRP synthesis. We chose to perform studies in the blood of patients with CAD as a model of chronic inflammation.

## 2. Experimental Section

### 2.1. Blood Samples Collection

Blood samples were collected from 14 male and female patients with CAD 46 ± 13 years old and 8 healthy volunteers 49 ± 13.6 years old. The clinical characteristic of patients, and inclusion and exclusion criteria are provided in Table S1 and the “treatment, inclusion and exclusion criteria’ section of the Supplementary Material. Blood was taken after at least eight hours of fasting from *vena cubitalis* in S-Monovette^TM^ vials (Sarstedt, Germany) with 3.2% sodium citrate at blood to anticoagulant ratio 1:9. Then, samples were transferred into separate tubes to perform the study in whole blood and platelet-poor plasma (PPP). PPP was prepared by centrifugation for 20 min at 2000 g. Samples were immediately prepared for flow cytometry as described further. Informed written consent was given by patients according to the Declaration of Helsinki, and the study was approved by the Ethics Committee of the National Medical Research Centre of Cardiology (Moscow, Russia).

### 2.2. The Antibody Panel for Detection of mCRP and pCRP Forms

To distinguish between mCRP and pCRP, we combined the FITC-labeled monoclonal antibody to pCRP (mAb to pCRP), clone 372 (ImTek, Moscow, Russia), and PE-labeled mAb to mCRP with cross-reactivity to pCRP (mAb to mCRP), clone 328 (ImTek, Moscow, Russia). The description of the production of these mAbs is provided in the Supplementary Material in the section “production of clones 372 and 328 of monoclonal antibodies to CRP”.

### 2.3. The Antibody Panels for Flow Cytometry

To detect different types of blood cells and their microparticles, we combined PE-Cy7-labeled mAb to CD235a, PerCP-Cy5.5-labeled mAb to CD45, APC-labeled mAb to CD41, APC-R700-labeled mAb to CD14, FITC-labeled mAb to CD63 (Becton Dickinson, Franklin Lakes, NJ, USA), V450-conjugated Annexin-V, and 1 µm latex beads (Thermo Fisher Scientific, Waltham, MA, USA) as a size reference. Exosomes were isolated with the Exo-FLOW^TM^ exosome capture kit and detected with EXOFITC^TM^ exosome stain (System Biosciences, Palo Alto, CA, USA) according to the manufacturer’s guide. As both the exosome stain and mAb to CD63 were FITC-labeled, we performed staining of each sample in separate tubes. We used a non-specific FITC-labeled mouse IgG as a negative control. In each test at least 10,000 events were registered.

### 2.4. Staining, Detection, and Counting of Cells and Microparticles in Blood Samples

Detection of cells, microparticles, and exosomes in samples were performed on the FACSCanto^TM^ II flow cytometer (Becton-Dickinson, Franklin Lakes, NJ, USA) on a day of sample collection. The threshold level of 200 units was chosen for Side Scatter to exclude debris and artifacts. Analysis of flow cytometry results was done on FACSDiva^TM^ software (Becton Dickinson, Franklin Lakes, NJ, USA). The same antibody panel was used to detect cells and microparticles. To calculate the number of microparticles per µL, we used BD Trucount^TM^ (Becton Dickinson, Franklin Lakes, NJ, USA) tubes as a reference.

### 2.5. Monocyte Culture Preparation

Monocytes were isolated and cultured according to a standard protocol. The detailed description is provided in the Supplementary Material in the section “monocyte culture preparation”. 

### 2.6. HepG2 Culture Preparation

The cell line of hepatocellular carcinoma of human HepG2 was kindly provided by P.N. Rutkevich (National Medical Research Center of Cardiology, Moscow, Russia). The detailed description is provided in the Supplementary Material in the section “HepG2 culture preparation”

### 2.7. Real-Time Polymerase Chain Reaction (RT-PCR)

Total RNA was isolated with the RNeasy^TM^ Mini Kit (Qiagen, Hilden, Germany) according to the manufacturer’s protocol. RNA concentrations were measured with a spectrophotometer Nanodrop (Thermo Fisher Scientific, Waltham, MA, USA) and quality was assessed by agarose gel electrophoresis. The obtained RNA in an amount of 3 μg was used to synthesize coding DNA (cDNA) with oligo(dT) primer using the Maxima^TM^ First Strand cDNA Synthesis Kit (Thermo Fisher Scientific, Waltham, MA, USA) according to the manufacturer’s protocol. PCR was performed with the SYBR^TM^ Green intercalating dye (Thermo Fisher Scientific, Waltham, MA, USA) in a Step One Plus™ Real-Time PCR System amplifier (Thermo Fisher Scientific, Waltham, MA, USA). As primers, unique pairs of oligodeoxynucleotides complementary to the analyzed mRNA/cDNA were used (Table S2 in the Supplementary Material). To avoid false-positive results, two pairs of CRP primers were used. The accession numbers for CRP primers are provided in Table S3 in the Supplementary Material. The reaction mixture (25 µL) contained 5 to 8 ng of cDNA and 10 pmol of primer in accordance with the standard protocol (Sintol, Moscow, Russia). The control mixture contained all components, with the exception of the matrix, replaced by de-ionized water. After the initial denaturation stage (950, 10 min), 40 amplification cycles were performed for all primer pairs with annealing and elongation at 600 °C for 60 s. The specificity of amplification was analyzed by melting the products after completion of the PCR, as well as by electrophoresis of amplicons in 1.5% agarose gel in a tris-acetate-EDTA (TAE) buffer (Thermo Fisher Scientific, Waltham, MA, USA).

### 2.8. Statistical Analysis

Data are presented as the mean ± standard deviation (mean ± SD). The Mann–Whitney U test was used to compare two groups of data and the Kruskal–Wallis ANOVA by ranks test to compare three or more samples. Differences were regarded as statistically significant if the null hypothesis was rejected with a probability >95%. The data were analyzed on the STATISTICA software v.6.0 (StatSoft Inc., Tulsa, OK, USA).

## 3. Results

**Figure 1:**
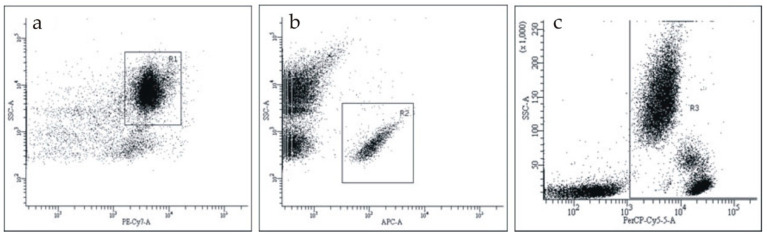
The gating of cells on the flow cytometry diagrams. (**a**) the gating of CD235a-PE-Cy7-positive events (erythrocytes), gate R1; (**b**) the gating of CD41-APC-positive events (platelets), gate R2; (**c**) the gating of CD45-PerCP-Cy5.5-positive events (leukocytes), gate R3. SSC-H—side scatter.

We used two antibodies to detect CRP: FITC-labeled mAb with verified selective reactivity to pCRP and PE-labeled mAb to mCRP with cross-reactivity to pCRP. The flow cytometry results, in which the signal from mAb to pCRP (FITC+/PE–) or both mAbs to pCRP and mCRP (FITC+/PE+) was present, were considered pCRP-positive. The flow cytometry results, in which the signal from mAb to pCRP was absent and the signal from mAb to mCRP was present (FITC–/PE+), were considered mCRP-positive (Figure S1 in the Supplementary Material). We studied CRP on the surface of blood cells in whole blood samples. The gating of each cell type is shown in Figure 1. To choose erythrocytes, we gated CD235a-PE-Cy7-positive events (Figure 1a); to choose platelets, we gated CD41-APC-positive events (Figure 1b); to choose leukocytes, we gated CD45-APC-positive events (Figure 1c).

**Figure 2:**
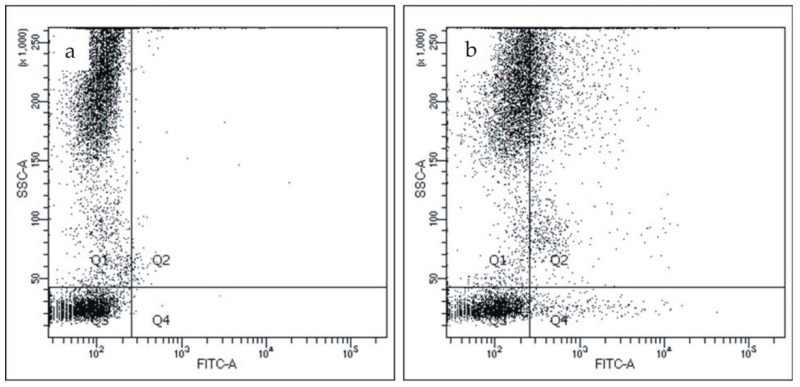
CRP detection on CD45-positive leukocytes. (**a**) The binding of the non-specific FITC-labeled mouse IgG to CD45-positive leukocytes; (**b**) the binding of FITC-labeled monoclonal antibody to pCRP to CD45-positive leukocytes. pCRP—pentameric C-reactive protein.

Then, we detected CRP on the surface of blood cells. As a negative control, we used the non-specific FITC-labeled mouse IgG. Figure 2b shows the binding of the FITC-labeled mAb to pCRP to CD45-positive leukocytes in comparison with the non-specific FITC-labeled mouse IgG (Figure 2a).

All obtained results were FITC+/PE+, meaning that pCRP, but not mCRP, was present on the surface of blood cells. As shown in Table 1, 4.3 ± 1.6% of erythrocytes, 3.5 ± 1.3% of platelets, and 26.5 ± 9.1% of leukocytes were pCRP-positive (*p* < 0.05, Kruskal–Wallis ANOVA by ranks test).

**Figure 3:**
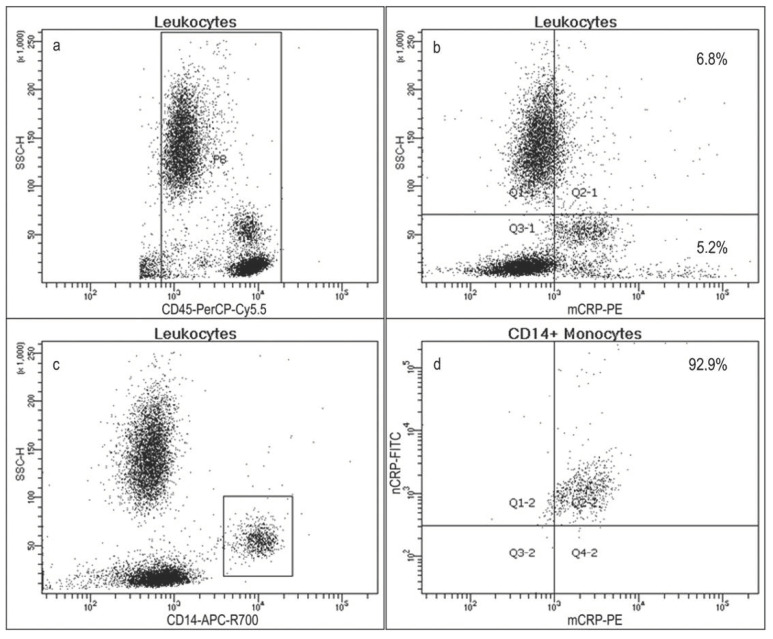
Flow cytometry diagrams of whole blood samples after erythrocyte lysis. (**a**) CD45-positive cells (leukocytes), selected with a rectangular gate; (**b**) mCRP-positive leukocytes; (**c**) CD14-positive leukocytes (monocytes), selected with a rectangular gate; (**d**) mCRP and pCRP-positive monocytes. pCRP—pentameric C-reactive protein; mCRP—monomeric C-reactive protein; SSC-H—side scatter.

We identified the subtype of pCRP-positive leukocytes. On flow cytometry diagrams, acquired after erythrocyte lysis, we selected CD45-positive leukocytes (Figure 3a). From this group, we selected a group of CRP-positive events (Figure 3b). These events comprised 12.0 ± 3.2% of the total population. The disposition of the densest group of events on the flow cytometry diagram fitted the typical pattern for monocytes. We verified this with monocyte-specific mAb to CD14 (Figure 3c). Monocytes comprised 7.3 ± 1.2% of all CD45-positive cells. As seen in Figure 3d, most monocytes were pCRP-positive (92.9 ± 6.8%). There were no cells, bearing mCRP.

**Figure 4:**
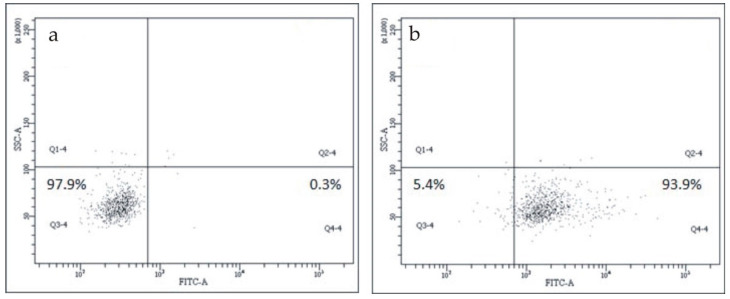
CRP detection on CD45/CD14-positive monocytes. (**a**) The binding of the non-specific FITC-labeled mouse IgG to CD14-positive monocytes; (**b**) the binding of FITC-labeled monoclonal antibody to pCRP to CD14-positive monocytes. pCRP—pentameric C-reactive protein.

Figure 4b shows the binding of the FITC-labeled mAb to pCRP to CD45/CD14-positive monocytes in comparison with the non-specific FITC-labeled mouse IgG (Figure 4a).

Next, we studied blood cell-derived microparticles in PPP. Following the established approach, we assumed that microvesicles are subcellular particles produced through outward blebbing of the plasma membrane of cells, and predominantly bear plasma membrane proteins; exosomes are particles produced in intracellular space, capturing cytoplasm content, including proteins, RNAs, and other intracellular entities [19]. We applied the same antibody panel as for cell detection. We added 1 µm latex beads into samples to verify that the size of detected events was smaller than 1 µm. Microvesicles contain phosphatidylserine in the outer membrane, which can be detected with annexin-V. To identify the membranous origin of microparticles, we used annexin-V, and to verify the intracellular origin of exosomes, we used ExoFITC^TM^ exosome capture kit and mAb to exosome-specific cytoskeletal protein CD63. As seen in Table 2, 64.1 ± 20.4% of erythrocyte microparticles, 0.5 ± 0.2% of platelet microparticles, and 12.9 ± 2.3% of leukocyte microparticles were pCRP-positive. Also, 21.1 ± 9.8% of erythrocyte microparticles, only 0.4 ± 0.3% of platelet microparticles, and as much as 47.9 ± 6.2% of leukocyte microparticles were pCRP-negative and mCRP-positive.

According to Annexin-V staining, most erythrocyte and a substantial part of platelet microparticles were Annexin-V positive microvesicles: 72.7 ± 11.9% of erythrocyte microparticles and 27.8 ± 6.4% of platelet microparticles, but only 3.5 ± 1.6 of leukocyte microparticles were annexin-V-positive (*p* < 0.05, Kruskal–Wallis ANOVA by ranks test). We isolated leukocyte microparticles with Exo-FLOW^TM^ exosome capture kit and then stained with exosome-specific stain ExoFITC^TM^. There was a distinct group of ExoFITC^TM^-positive, mCRP-positive exosomes (22.0 ± 9.6%). To double-check our result, we stained PPP samples with mAb to exosome-specific cytoskeletal protein CD63: 20.8 ± 11.2% of microparticles were CD63-positive, mCRP-positive.

We measured mCRP-positive CD45-positive microparticle counts in the blood of 14 patients with CAD and 8 healthy volunteers. In the blood of patients with CAD, mCRP-positive CD45-positive microparticle counts were higher (8764 ± 2876 particles per µL, *n* = 14) than in the blood of healthy volunteers (1472 ± 307 particles per µL, *n* = 8). The difference between the results was statistically significant (*p* < 0.05, Mann–Whitney U test). This study did not investigate the relationship between the severity of CAD and CRP-positive cells or microparticle numbers.

**Figure 5:**
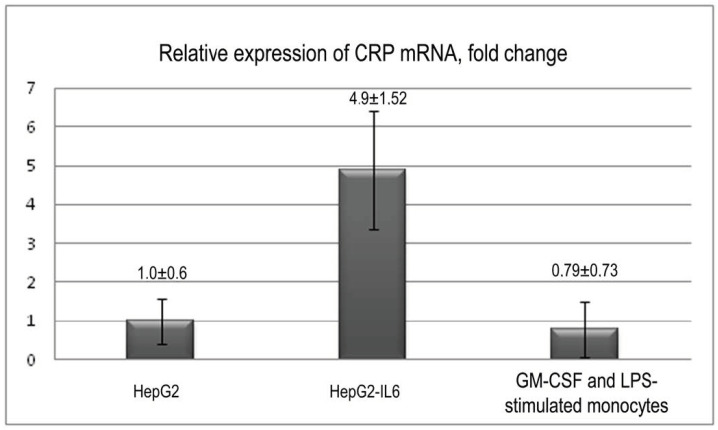
Real-time polymerase chain reaction (RT-PCR) results of the relative expression of CRP. CRP—C-reactive protein. HepG2—human cell line of hepatocellular carcinoma. HepG2-IL6—interleukin-6-stimulated HepG2 cells. GM-CSF, LPS-stimulated monocytes—granulocyte-macrophage colony-stimulating factor and lipopolysaccharide-stimulated peripheral blood monocytes. CRP expression was normalized against β-actin mRNA expression. Data expressed as values relative to the HepG2 group. Error bars represent the mean ± SD.

The expression of CRP was quantified with RT-PCR in LPS, GM-CSF stimulated monocytes, IL-6-stimulated and unstimulated hepatocytes of the HepG2 cell line. The data presented as the relation of the expression of CRP mRNA in stimulated monocytes and stimulated HepG2 hepatocytes to the expression in unstimulated HepG2 hepatocytes. As shown in Figure 5, which presents the combined results of PCR from two CRP primers used in three independent experiments, the expression of CRP mRNA in LPS and GM-CSF-stimulated monocytes was 0.79 ± 0.73-fold, which is slightly lower relative to HepG2, and approximately 6-fold lower, than in IL-6 stimulated HepG2 (4.9 ± 1.52-fold), but is still detectable (*p* < 0.05, Kruskal–Wallis ANOVA by ranks test). mRNA levels of CRP between probes were normalized by the mRNA levels of β-actin as a housekeeping gene. 

**Figure 6:**
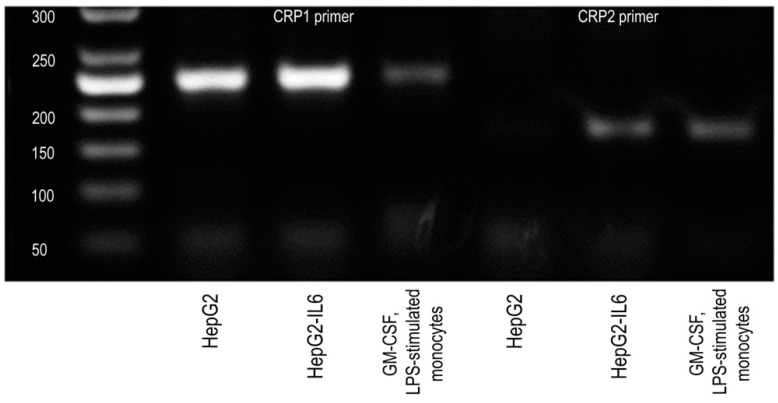
Electropherogram of PCR fragments in 1.5% agarose gel in a tris-acetate-EDTA buffer. Scale to the left: the corresponding number of base pairs. CRP expression was detected in: HepG2—human cell line of hepatocellular carcinoma; HepG2-IL6—interleukin-6-stimulated HepG2 cells; GM-CSF, LPS-stimulated monocytes—granulocyte-macrophage colony-stimulating factor and lipopolysaccharide-stimulated peripheral blood monocytes; b.p.—base pairs.

In addition, CRP mRNA expression was validated by electrophoresis of amplicons in 1.5% agarose gel. The sizes of the PCR products that corresponded to CRP mRNA were 167 and 245 base pairs. The PCR product for the housekeeping gene ActB was 144 bp. Both CRP transcripts (245 base pairs and 167 base pairs) were detected at low levels in stimulated monocytes (Figure 6). CRP transcripts from HepG2 and HepG2 stimulated with IL-6 were used as a positive control. Thus, monocytes stimulated with LPS and GM-CSF express CRP mRNA transcripts at a low but detectable level, which means that they are able to express CRP mRNA.

## 4. Discussion

There is growing evidence that mCRP can be detected in circulating blood. Habersberger et al. identified mCRP on microparticles circulating in the blood of persons with acute myocardial infarction (AMI) [17]. Later, Crawford et al. described mCRP on circulating endothelial microparticles in patients with peripheral artery disease [18]. Wang et al. reported a significant increase in the concentration of monomeric CRP in the circulating blood of patients with AMI. The increase in mCRP concentration was also detected in patients with elevated pCRP concentration in plasma, unrelated to ischemic myocardial damage [20]. Another study reported a positive correlation between the rise in mCRP levels and troponin I in AMI [21].

Taking into consideration the evidence that mCRP can be detected in circulating blood, particularly on microparticles, we studied blood cells and microparticles to identify which of them bear mCRP. Monocytes had the largest population of CRP-bearing cells. These cells presented a pentameric, but not monomeric form of CRP. The presence of pCRP on monocyte membranes may facilitate the process of dissociation that occurs in vivo [4,12]. In the case of dissociation, CRP on monocytes can possibly be involved in the modulation of local inflammatory response.

We also detected pCRP on a substantial number of circulating cell-derived microparticles, mainly on Annexin-V positive microvesicles, and mCRP on annexin-V negative leukocyte-derived exosomes. Microvesicles are fragments of cell membranes, shed upon cell activation. Microvesicles are much smaller than cells, with size usually varying from 0.1 to 1 µm in diameter. In the circulation, they bear signal molecules and have antigen-presenting capabilities. They are involved in immunomodulation as either pro-inflammatory or anti-inflammatory agents [22,23]. Microvesicles are highly procoagulant since they present negatively charged phospholipids involved in the assembly of components of the coagulation cascade [24]. Also, the presence of negatively charged phospholipids, especially phosphatidylcholine, is a prerequisite for pCRP dissociation into mCRP subunits [12,25].

Exosomes belong to another group of cell-derived microparticles. Exosomes are usually smaller than microvesicles, with a size of less than 0.2 µm. Unlike microvesicles, which originate from cell membranes, exosomes are produced in the endocytic-lysosomal system of cells and released by exocytosis. Exosome cargo can contain proteins, RNA, and other molecules of intracellular origin. In the blood flow, exosomes may act as transporters for this potentially regulatory cargo to target cells [19]. Accumulated data demonstrate the involvement of exosomes in vascular dysfunction and the development of inflammation [26].

The emerging data show that mononuclear cells are able to transport other regulating proteins in CAD as well, such as the adenosine A2A receptor [27]. This signaling receptor associated with myocardial ischemia was found in extracellular vesicles isolated from plasma of patients with CAD and culture supernatant of stimulated human T cells line [28], as well as on peripheral blood mononuclear cells isolated from patients with CAD [29].

Previously, we reported higher counts of mCRP-positive leukocyte-derived microparticles in the blood of patients with CAD, compared to healthy volunteers [30]. In this study, we compared the counts of mCRP-positive leukocyte-derived microparticles in the blood of 14 patients with CAD with those of 8 healthy volunteers. In patients with CAD, mCRP-positive leukocyte derived microparticle counts were significantly higher than in practically healthy volunteers. The obtained results closely correlate with our previous data. We speculate that higher counts of mCRP-positive microparticles of leukocyte origin in the blood of patients with CAD may indicate the involvement of mCRP in the maintenance of the inflammatory response.

We studied the expression of CRP mRNA in LPS and GM-CSF-stimulated monocytes isolated from human peripheral blood. The level of expression was slightly lower relative to HepG2 but still detectable. We hypothesize that in vivo recruited monocytes/macrophages, stimulated in areas of local inflammation, may synthesize CRP directly in monomeric form, or in pentameric form, which may then undergo dissociation. Being presented on the membrane surface or shed with microparticles, it may influence the inflammatory response. This hypothesis is in line with the existing data. We have already mentioned papers that describe the expression of CRP mRNA by monocyte-derived macrophages [13], alveolar macrophages [14], mCRP expression by human peripheral blood monocytes [15], and mCRP production by the U937 line of macrophages [16]. Also, CRP mRNA expression and CRP synthesis in small amounts was observed ex vivo on LPS-stimulated peripheral blood monocytes [31]. Local synthesis of monomeric CRP subunits by some subpopulations of lymphocytes was described by Kuta and Baum [32]. The finding that activated monocytes are able to express CRP mRNA indicates that monocytes might not be just transporters of CRP. We did not study whether monocytes can synthesize CRP. This may be an interesting direction for future research, considering the known roles of monocytes and mCRP in the development of local inflammation.

## 5. Conclusions

Our results show that pCRP does not exist in blood flow only in a soluble form, but intensively binds to blood cells, and appear on their microparticles. Among blood cells, leukocytes had the largest pCRP-positive population (up to 26.5 ± 9.1% of all leukocytes); 92.9 ± 6.8% of monocytes were pCRP-positive. mCRP presence was less evident, except for a large group of monocyte-derived exosomes, which comprised up to 22.0 ± 9.6% of the whole population. We found that GM-CSF and LPS-stimulated monocytes expressed CRP transcripts at a low but detectable level, which means that they are able to express CRP mRNA. In the blood of patients with CAD, mCRP-positive CD45-positive microparticle counts were significantly higher (8764 ± 2876 particles per µL, *n* = 14), than in the blood of healthy volunteers (1472 ± 307 particles per µL, *n* = 8). CRP mRNA expression by monocytes, the presence of mCRP on monocyte-derived exosomes and higher counts of mCRP-positive leukocyte-derived microparticles in the blood of patients with CAD, compared to healthy volunteers, may indicate the involvement of mCRP in the maintenance of the inflammatory response.

## 6. Patents

The method used in this work for the measurement of monomeric CRP with a combination of selective mAb to pCRP and cross-reactive mAb to mCRP and pCRP was patented in 2019. Patent RU2704128C1 “Method for measuring concentration of monomer C-reactive protein on the surface of blood cells”.

## Figures and Tables

**Table 1:** CRP-positive cells.

	CRP-Positive Cells, %
CD235a-positive erythrocytes	4.3 ± 1.6
CD41-positive platelets	3.5 ± 1.3
CD45-positive leukocytes	26.5 ± 9.1

**Table 2:** CRP-positive microparticles.

	mCRP-Positive MPs, %	pCRP-Positive MPs, %	*p* *
Erythrocyte-derived MPs	21.1 ± 9.8	64.1 ± 20.4	*p* > 0.05
Platelet-derived MPs	0.4 ± 0.3	0.5 ± 0.2	*p* > 0.05
Leukocyte-derived MPs	47.9 ± 6.2	12.9 ± 2.3	*p* < 0.05

MPs—microparticles. * Mann–Whitney U test. mCRP—monomeric C-reactive protein; pCRP—pentameric C-reactive protein.

## Supplementary Materials

The following are available online at https://www.mdpi.com/2227-9059/8/10/435/s1, Table S1: Clinical characteristic of the CAD patients, Table S2: Primers used for RT-PCR, Table S3: The accession numbers for CRP primers, Figure S1: A schematic representation of the interaction of monoclonal antibodies to mCRP and pCRP with their ligands.
