# Supplementary material for: CRP Is Transported by Monocytes and Monocyte-Derived Exosomes in the Blood of Patients with Coronary Artery Disease
Source: Biomedicines. 2020 Oct 19;8(10):435. doi: 10.3390/biomedicines8100435 (PMC7589628; doi:10.3390/biomedicines8100435)
Supplement: Supplementary file 1 [file biomedicines-08-00435-s001.pdf]

biomedicines

Supplementary Material

CRP Is Transported by Monocytes and Monocyte-Derived Exosomes in the Blood of Patients with Coronary Artery Disease

1. Clinical characteristic of the CAD patients

Table S1: Clinical characteristic of the CAD patients

	CAD (n=14)
**Age, years**	46±13
**Men/Women**	12 (86%)/2(14%)
**History of myocardial infarction**	9 (64%)
**Body mass index (kg/m²)**	27±4.2
**Arterial Hypertension**	12 (86%)
**Diabetes mellitus**	2 (14%)
**Total cholesterol (mmol/L)**	5.1±0.7
**LDL-cholesterol (mmol/L)**	3.3±0.6
**HDL-cholesterol (mmol/L)**	1.1±0.2
**Triglycerides (mmol/L)**	1.8±0.6
**Currently smokers**	11 (76%)
**Family history of CAD**	8 (57%)
**One-vessel disease**	5(36%)
**Two-vessel disease**	7 (50%)
**Triple-vessel disease**	2 (14%)

2. Treatment, inclusion and exclusion criteria

Inclusion criteria: patients with coronary artery disease, verified by coronary angiography, in the age of 18-70.

A total of 14 consecutive male and female patients 46±13 years old who had been referred to the department of atherosclerosis for the assessment of CAD were included in the present study. The inclusion criteria were angiographically documented: (≥50% stenosis of one or more of the major epicardial coronary arteries). The results of the measurement of mCRP-positive leukocyte-derived microparticle counts were compared with those of 8 practically healthy control male and female subjects matched for age who were recruited from hospital staff and their relatives. All of them had normal ECG patterns, normal findings of ultrasound examination of the heart, and negative treadmill test results. Those with unstable angina, myocardial infarction, congestive heart failure, any known inflammatory or autoimmune disease, acute or chronic inflammation and/or immunomodulatory medication were excluded from the study.

Exclusion criteria: unstable angina, myocardial infarction, surgical intervention within last 60 days, drug-eluting stent implantation in the previous 12 months, stroke, congestive heart failure, renal failure (GFR<60ml/min/1.73m^2 or serum creatinine 150 μMol/l), liver failure (ALT or AST more than 3 fold above upper normal limit), decompensated diabetes mellitus (fasting plasma glucose > 7 mMol/l or plasma glucose > 11.1 mMol/l on any measurement), acute inflammation, chronic autoimmune or infectious diseases and/or immunomodulatory medications, malignant tumors, allergic reactions (eosinophilia > 700 cells/μL).

Treatment: All patients received aspirin, statins, β-adrenoblockers and angiotensin-converting enzyme (ACE) inhibitors or angiotensin receptor antagonists. Other antihypertensive drugs were prescribed according to physician decision. One patient was prescribed metformin due to diabetes mellitus.

Clinical characteristics of the study patients are summarized in the Table S1.

3. Production of clones 372 and 328 of monoclonal antibodies to CRP

Animal handling and use complied with the Guide for the Care and Use of Laboratory Animals published by the US National Institutes of Health [1]. Mice (strain Balb/c) were immunized by either pentameric or denaturized human CRP in emulsion of Complete Freund's adjuvant and then incomplete Freund's adjuvant (ImTek, Russia). Hybridomas producing monoclonal antibody against CRP were developed according to the standard protocol [2]. Screening of hybridomas secreting IgG specific to human CRP into the culture media was performed with ELISA with the antigen immobilized in carbonate buffer, pH 9.6. Eight different clones were used as the source of antibodies produced in the ascitic fluids of Balb/c mice. Monoclonal antibodies to CRP were purified using affinity chromatography on protein G immobilized on sepharose. Two antibodies, MOH pCRP (clone 372) and MOH mCRP (clone 328) were selected for their pronounced specific reaction to human CRP in the pentameric or monomeric form, respectively. So as pentameric CRP directly adsorbed on the plastic presents both "pentameric" and "denaturized" epitopes, sandwich ELISA with immobilized (capture) rabbit affinity purified antibody to CRP (ImTek, Russia) was used to demonstrate selective reaction to pCRP vs mCRP. Reduced and non-reduced SDS-PAGE followed by Western blotting staining with MOH pCRP and MOH mCRP was used for further characterization of their specificity. Human pCRP (ImTek, Russia), purified from human pleural fluid as earlier described [3] was used for preparation and characterization of the antibodies. mCRP

was prepared according to the method of Potempa et al. [4]. Solution of the pCRP (1 mg/ml) was incubated in 8 M urea, 0.01 M EDTA for 3 h at 37°C, followed by dialysis against 0.01 M Tris-Cl, 0.05 M NaCl, pH 7.5.

4. Monocyte culture preparation

Mononuclear cells were isolated by centrifugation in Ficoll-verographin DIACOLL density gradient (Dia-M, Russia), density=1.077g/ml, according to the method of Böyum [5]. Isolated cells were seeded into wells of a 24-well plate at a concentration of $2x10^6$/ml in Roswell Park Memorial Institute (RPMI) 1640 medium (Thermo Fischer, USA) supplemented with 10% fetal bovine serum (FBS) (Thermo Fischer, USA), 2 mM L-glutamine (Thermo Fischer, USA), 50 U/ml penicillin and streptomycin (Thermo Fischer, USA). After 1 hour, the unattached cells were washed, and the attached cells were cultured in the same medium with the addition of 10 ng/ml granulocyte-macrophage colony-stimulating factor (GM-CSF) (Myltenyi Biotech, Germany) at 37°C in an atmosphere of 5% CO_2. After 2 days, part of the used medium was changed to fresh medium with the addition of GM-CSF. After 3 days, the medium was replaced with EX VIVO-15™ (Lonza, Switzerland) with the addition of 1 mM sodium pyruvate, 2 mM L-glutamine (Thermo Fischer, USA) and minimum essential medium (MEM) amino acids solution at 1:100 ratio (Thermo Fischer, USA). After 48 hours, 100 ng/ml LPS (Sigma-Aldrich, USA) was added for 24 hours.

5. HepG2 culture preparation

Cells were cultured in Dulbecco's Monomeric Eagle Medium (DMEM) (Thermo Fischer, USA) with glucose content of 1 g/l, 10% FBS (Thermo Fischer, USA), and 100 µM of MEM amino acids solution (Thermo Fischer, USA). Two passages after thawing, the cells were stimulated by addition of 20 ng/ml of recombinant IL-6 (SCI-Store, Russia) to the culture medium. After 18 hours, the cells were washed with phosphate buffered saline (PBS) (Dia-M, Russia) and lysed for the subsequent isolation of RNA.

6. Primers used for CRP and their accession numbers

Table S2: Primers used for RT-PCR

Gene	Sense primer	Antisense primer	Amplicon (b.p.)	Template
β -actin	5′- CCTGGCACCCAGCACAAT-3′	5′- GGGCCGGACTCGTCATAC -3′	144	1149-1292
CRP-1	5′- TCAAAGCCTTCACTGTGTGC-3′	5'- TACCCAGAACTCCACGATCC-3′	245	247-491
CRP-2	5′- GTCTTGACCAGCCTCTCTCA-3	5′- GTCGAGGACAGTTCCGTGTA-3′	167	129-295

RT-PCR – real-time polymerase chain reaction; CRP – C-reactive protein; b.p. – base pairs.

Table S3: The accession numbers for CRP primers

	CRP-1 primer	CRP-2 primer

Transcript variant 1	NM_001329057.2	NM_001329057.2
Transcript variant 2	NM_000567.3	NM_000567.3
Transcript variant 3	n/a	NM_001329058.2
Transcript variant 4	n/a	NM_001382703.1

7. A schematic representation of the interaction of monoclonal antibodies to mCRP and pCRP with their ligands

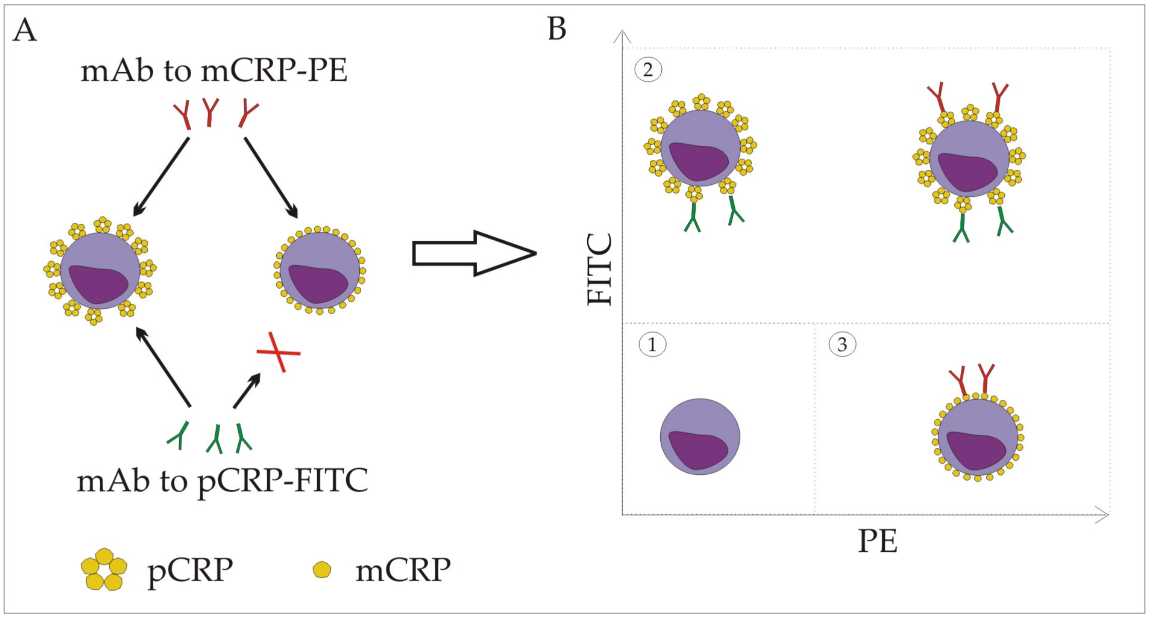

Figure S1: A schematic representation of the interaction of monoclonal antibodies to mCRP and pCRP with their ligands. A) a schematic representation of the interaction of mAbs to mCRP and pCRP with their ligands; B) a representation of FACS diagram with the possible distribution of the registered events. 1) CRP-negative events; 2) pCRP-positive events; 3) mCRP positive events. mAb to mCRP is PE-labeled, mAb to pCRP is FITC-labeled. mAb - monoclonal antibody; pCRP - pentameric C-reactive protein; mCRP - monomeric C-reactive protein.

References

1. National Research Council (US) Committee for the Update of the Guide for the Care and Use of Laboratory Animals. Guide for the Care and Use of Laboratory Animals, 8th ed. National Academies Press (US): Washington (DC), USA, 2011; doi: 10.17226/12910.
2. Nakazawa, M.; Mukumoto, M.; Miyatake K. Production and purification of monoclonal antibodies. *Methods Mol Biol.* **2010**, *657*, 75–91; doi: 10.1007/978-1-60761-783-9_6.
3. Hashimoto, K.; Tatsumi, N. Rapid isolation of human C reactive protein and serum amyloid P component. *J. Immunol. Methods.* **1989**, 125, 295–296.
4. Kresl, J.J.; Potempa, L.A.; Anderson, B.E. Conversion of native oligomeric to a monomeric monomeric form of human C-reactive protein. *Int. J. Biochem. Cell Biol.* **1998**, *30*, 1415–1426.
5. Böyum, A. Isolation of mononuclear cells and granulocytes from human blood. Isolation of monuclear cells by one centrifugation, and of granulocytes by combining centrifugation and sedimentation at 1 g. *Scand. J. Clin. Lab. Invest. Suppl.* **1968**, *97*, 77–89.
